# Model and Non-model Insects in Chronobiology

**DOI:** 10.3389/fnbeh.2020.601676

**Published:** 2020-11-26

**Authors:** Katharina Beer, Charlotte Helfrich-Förster

**Affiliations:** Neurobiology and Genetics, Theodor-Boveri Institute, Biocentre, Am Hubland, University of Würzburg, Würzburg, Germany

**Keywords:** *Drosophila melanogaster*, *Apis mellifera*, circadian clock, complex behavior, diapause, sociality

## Abstract

The fruit fly *Drosophila melanogaster* is an established model organism in chronobiology, because genetic manipulation and breeding in the laboratory are easy. The circadian clock neuroanatomy in *D. melanogaster* is one of the best-known clock networks in insects and basic circadian behavior has been characterized in detail in this insect. Another model in chronobiology is the honey bee *Apis mellifera*, of which diurnal foraging behavior has been described already in the early twentieth century. *A. mellifera* hallmarks the research on the interplay between the clock and sociality and complex behaviors like sun compass navigation and time-place-learning. Nevertheless, there are aspects of clock structure and function, like for example the role of the clock in photoperiodism and diapause, which can be only insufficiently investigated in these two models. Unlike high-latitude flies such as *Chymomyza costata* or *D. ezoana*, cosmopolitan *D. melanogaster* flies do not display a photoperiodic diapause. Similarly, *A. mellifera* bees do not go into “real” diapause, but most solitary bee species exhibit an obligatory diapause. Furthermore, sociality evolved in different Hymenoptera independently, wherefore it might be misleading to study the social clock only in one social insect. Consequently, additional research on non-model insects is required to understand the circadian clock in Diptera and Hymenoptera. In this review, we introduce the two chronobiology model insects *D. melanogaster* and *A. mellifera*, compare them with other insects and show their advantages and limitations as general models for insect circadian clocks.

## Introduction: The History of Insect Models in Chronobiology

Chronobiology is a field of biology that examines cyclic phenomena in living organisms and their adaptation to solar- and lunar-related rhythms. These cycles are known as biological rhythms and the best known are daily, annual and lunar rhythms. Daily rhythms are controlled by the circadian clock, which has a period of about (circa) a day (dian), but is synchronized to a period of 24 h by the environmental rhythms (= Zeitgeber) on earth. Chronobiologists also say that the circadian clock entrains to the 24 h Zeitgeber. The circadian clock is ubiquitous in living organisms of our planet. Circadian clocks help individual insects and other organisms to anticipate the 24 h environmental cycles and insect populations to synchronize crucial steps in their life (such as eclosion from the pupal case or mating) at the optimal time of the day. In addition, they enable individual insects to measure time, which is important for a time memory. They also provide an internal time reference for insects that orient themselves via a sun compass, which is necessary to compensate for the sun's predictable daily motion. Furthermore, the circadian clock is needed to measure day length and to prepare in time for the coming season (e.g., to reproduce or to hibernate). Since insects live at different latitudes (from the equator to the poles), at different habitats (conditions of the surrounding environment) and have developed different life styles (e.g., solitary or social), several adaptations of the circadian clock evolved that are just beginning to be investigated.

Different insect models help to elucidate various aspects of circadian clock function. Nevertheless, many concepts in chronobiology, like the interplay of daily and annual time keeping in photoperiodism and hibernation (in insects called diapause) or the influence of inter-individual behavior and social insect communities on the clock, are still not well-understood.

The honey bee was one of the first insect models in chronobiology. Reports of daily foraging behavior in the beginning of the twentieth century inspired research on the biological relevance of clock regulated behavior (Kleber, 1935; Galizia et al., 2011). Studies on the role of the clock in complex behaviors like sun-compass orientation of the honey bee followed (Frisch and Lindauer, 1954; Medugorac and Lindauer, 1967; Lehmann et al., 2011; Cheeseman et al., 2012) and are still an intensely studied topic, since many other insects of different orders, for example the monarch butterfly, desert locusts and the desert ant *Cataglyphis*, use sun- or sky-compass orientation (e.g., Fent and Wehner, 1985; Merlin et al., 2009, 2012; Homberg et al., 2011; Homberg, 2015).

On the search for the location of the circadian clock in the insect brain, first evidence of a circadian pacemaker (= master clock) in the lateral brain was given by surgical removal and transplantation of the optic lobes in cockroaches (Nishiitsutsuji-Uwo and Pittendrigh, 1968; Page, 1982). Later, more specific tissue transplantation studies identified the accessory medulla, a small neuropil in the optic lobe, as the master clock in cockroaches (Reischig and Stengl, 2003). In comparative studies, master clocks in the lateral and/or dorsal brain could be identified in many different species, for example flies, bugs, bees and some moth species (Siwicki et al., 1988; Nässel et al., 1993; Helfrich-Förster et al., 1998; Wise et al., 2002; Závodská et al., 2003; Vafopoulou et al., 2009; Ikeno et al., 2014; Kobelková et al., 2015; Fuchikawa et al., 2017).

With the isolation of the first clock gene mutants in *Drosophila melanogaster* the molecular basis of the circadian clock was unraveled and subsequently the first functional studies were introduced to insect chronobiology (Konopka and Benzer, 1971). Systematic genetic manipulations of the clock system led to a detailed knowledge about the insect clock in this fly [reviewed in (Hall, 2003)] and therefore the best description of basic concepts of the insect circadian clock so far is found in *Drosophila*.

Lately, new arising methods for genetic manipulation offer the possibility to study circadian clock components and function in detail also in many other insects. RNA interference has been successfully applied in different insects (Moriyama et al., 2008; Lee et al., 2009; for example: Ikeno et al., 2010; Takekata et al., 2012; Kotwica-Rolinska et al., 2017) and genome editing via CRISPR (Clustered Regularly Interspaced Short Palindromic Repeats)—Cas (CRISPR associated protein) may provide clock gene manipulation suitable for further insects (Kotwica-Rolinska et al., 2019).

## The Molecular Clock—the Central Negative Feedback Loop

**Figure 1:**
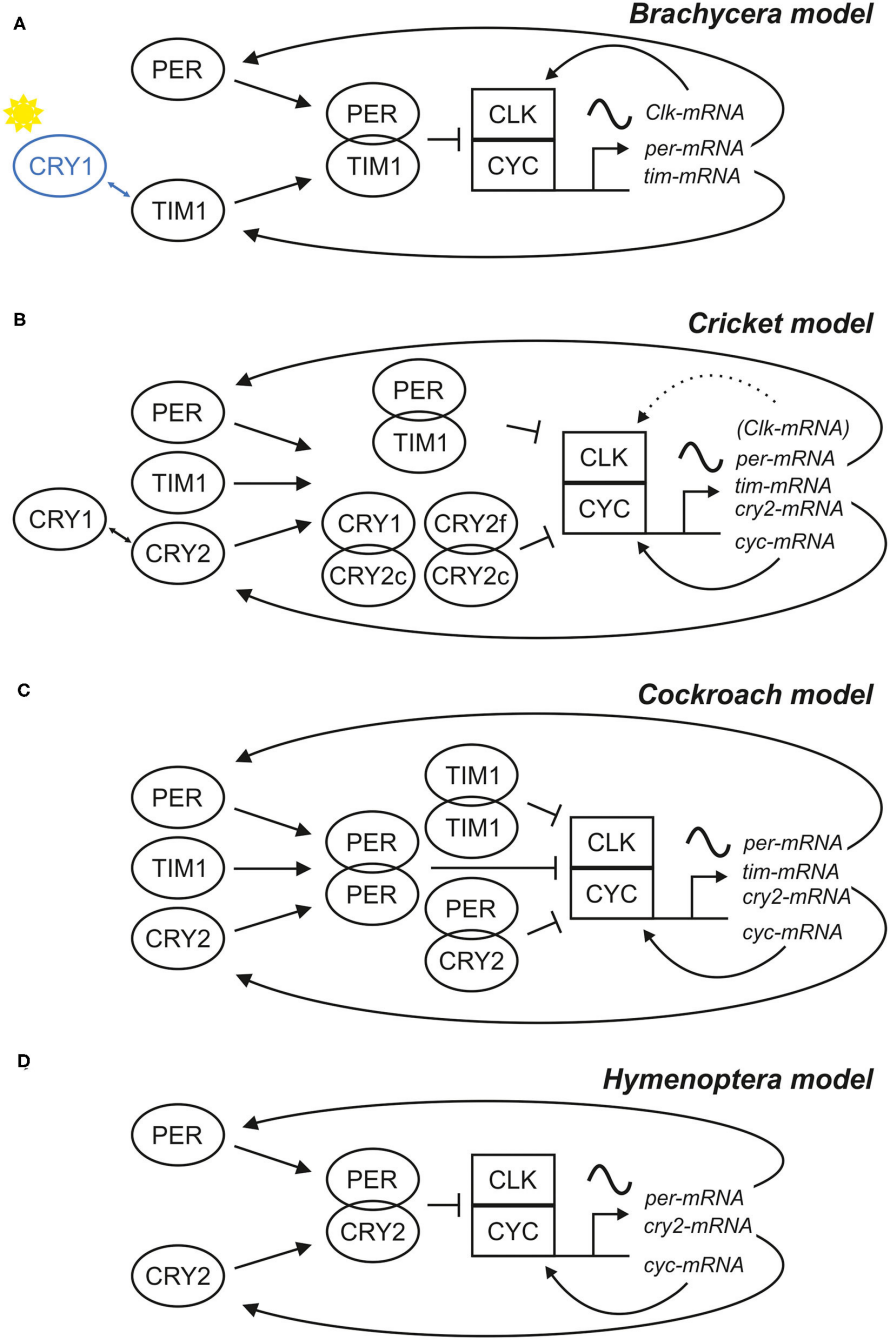
Scheme of four different models for the central negative feedback loop(s). Models based on different clock gene sets have been described for **(A)** *Drosophila* and other **Brachycera**, **(B)** the **cricket** *Gryllus bimaculatus*, **(C)** the **cockroach** *Rhyoparobia maderae* and **(D)** the honey bee *Apis mellifera* and other **Hymenoptera**. Different sets of clock proteins (left side of the schemes) build different combinations of dimers, which regulate the cyclic expression of clock gene mRNAs, which in turn provide the basis of clock protein production. In all four systems, the rhythms are generated by a negative feedback of some clock proteins on their transcriptional activators CLK/CYC, but there are differences in the composition of these clock proteins and their properties. In Brachycera, CRY1 acts as a blue-light receptor and can bind TIM1 **(A)**, causing degradation of TIM1 in a light dependent manner. In contrast, the cricket relies purely on photoreception in the compound eye and CRY1 acts together with different isoforms of CRY2 (CRY2c and CRY2f) as part of a second central negative feedback loop **(B)**. In all models, except for the Brachycera model, *cyc* was expressed rhythmically and *Clk* constitutively. Nonetheless, *cyc* knock-down studies showed, that *Clk* expression cycles in the absence of CYC, which implies that *Clk* expression is rhythmically regulated in crickets and rhythms are masked in the natural state (Uryu et al., 2013). It is highly likely that more than four different insect clock models for the central negative feedback loop exist, because in sand flies for example, both *Clk* and *cyc* are rhythmically expressed (Meireles-Filho et al., 2006a,b; Meireles-Filho and Kyriacou, 2013).

In 2017, the Nobel Prize in Physiology/Medicine was awarded to Jeffrey Hall, Michael Young, and Michael Rosbash for their work that led to the understanding of the molecular basis of circadian rhythms in *D. melanogaster*, a work that was pioneered by Konopka and Benzer in the 70ties of the last century by the isolation of the *period* mutants (Konopka and Benzer, 1971). The *period* gene (*per*) was the first clock gene that was ever isolated and it turned out to be highly conserved in the animal kingdom (Table 1). Similarly conserved are the general mechanisms of molecular rhythm generation that involve several other clock genes and proteins that interact in transcriptional/translational feedback loops. Nevertheless, a few features are unique to *D. melanogaster*, or better to say to higher flies (Brachycera) (Sandrelli et al., 2008; Tomioka and Matsumoto, 2015; Chahad-Ehlers, 2017; Bertolini et al., 2018). For example, the second discovered fly clock gene, *timeless1* (*tim1 or dtim*) has a unique function in the first transcriptional/translational feedback loop of higher flies, where its protein product TIM1 dimerizes with PER (the protein product of the *period* gene) and the dimer enters the nucleus (Sehgal et al., 1994; Myers et al., 1996; Saez and Young, 1996) (see Figure 1). In other animals, for example the honey bee, *tim1* is substituted by a *cryptochrome* (*cry*) *gene* that codes for a specific light-insensitive form of CRY2 also called mammalian type CRY (mCRY) (Yuan et al., 2007). Another *cry* gene [*Drosophila cry* (*dcry*) or *insect type cry1* (*cry1*)] codes for a light-sensitive CRY1 and usually forms no dimers with PER (Emery et al., 1998), although PER-CRY1 interactions have been found *in vitro* (Rosato et al., 2001; Schlichting et al., 2018). In the fruit fly, CRY1 interacts with TIM1 (insect type TIM1) after it has been activated by light and leads to the degradation of TIM1 in the proteasome (Ceriani et al., 1999; Naidoo, 1999), a feature that makes flies very sensitive to light (see below). TIM1 and CRY1 are also present in for example mosquitoes, aphids, crickets, butter flies and moths (Iwai et al., 2006; Gentile et al., 2009; Cortés et al., 2010; Danbara et al., 2010; Kobelková et al., 2015; Rodriguez-Sanchez et al., 2015; Shaikevich et al., 2016; Barberà et al., 2017). However, in these insects they exist in addition to CRY2 (insect CRY2 or mammalian type CRY) and it is not completely clear, whether and how TIM1 interacts with PER. *Tim1* knock-down studies in crickets gave evidence that indeed *tim1* seems not essential for the central feedback mechanism in crickets, because the knock-down did not destroy rhythmic behavior in the animals (Danbara et al., 2010). Following knock-down studies revealed that circadian behavior of crickets is maintained when either *cry2* or *tim* are rhythmically expressed and that there appear to exist two interconnected negative feedback loops, in which *cry1* and *cry2* apart from *per* and *tim1* are important to maintain clock function (Tokuoka et al., 2017) (see Figure 1). Unlike in *Drosophila*, CRY1 does not act as a blue light photoreceptor and light entrainment in the cricket relies purely on photoreception via the compound eyes (Komada et al., 2015; Kutaragi et al., 2018). In other species that lack *cry1* (i.e., beetles and cockroaches), *tim1* may play a different role in the circadian rhythm generation (Werckenthin et al., 2012, 2020; Li C-J et al., 2018), which stresses the complexity in evolution of divers sets of clock genes in different insects. Recently, a model which includes two different pacemakers with different clock gene sets was proposed for the cockroach to explain *per, cry2*, and *tim1* function in parallel (Werckenthin et al., 2020) (see Figure 1). The cockroach clock may work with three regulatory loops, because knock-down of neither *per*, nor *tim1* nor *cry2* alone was successful to induce arrhythmic behavior in the animals (Werckenthin et al., 2020). Interestingly, Hymenoptera are so far the only insect group that lack both, *cry1* and *tim1* (only *cry2* and *tim2* are present; see also section “***The relevance of Zeitgebers differs*** ***between flies and bees***”), and they display a clock gene set that is more similar to the mammalian clock (Rubin et al., 2006; Sandrelli et al., 2008). In the following, we will describe the principal transcriptional/translational feedback loops that lead to circadian oscillations with a focus on Diptera and Hymenoptera (see for more insight: Hardin, 2011; Brown et al., 2012; Özkaya and Rosato, 2012; Hardin and Panda, 2013; Helfrich-Förster, 2017; Top and Young, 2018).

**Table 1:** Different sets of clock components in insects.

**Insect model**	**PER**	**CRY1**	**CRY2**	**TIM1**	**TIM2**	**CLK**	**CYC**	**PDP1**	**VRI**	**CWO**	**JET**	**PDF**	**References**
*Drosophila melanogaster*	√	√	x	√	√	√	√	√	√	√	√	√	Brown et al., 2012; Tomioka and Matsumoto, 2015
*Anopheles gambiae*	√	√	√	√	√	√	√	√	√	√	?	√	Janssen et al., 2008; Ingram et al., 2012; Tomioka and Matsumoto, 2015
*Danaus plexippus*	√	√	√	√	√	√	√	√	√	√	?	√	Zhu et al., 2005, 2008; Reppert et al., 2016; Lam and Chiu, 2019
*Gryllus bimaculatus*	√	√	√	√	√	√	√	√	√	?	?	√	Singaravel et al., 2003; Moriyama et al., 2008, 2012; Danbara et al., 2010; Hassaneen et al., 2011; Uryu et al., 2013; Tokuoka et al., 2017; Nose et al., 2018; Narasaki-Funo et al., 2020
*Acyrthosiphon pisum*	√	√	√	√	√	√	√	√	√	√	x	x^*^	Cortés et al., 2010; Barberà et al., 2017; Lam and Chiu, 2019
*Rhyparobia maderae*	√	x	√	√	√	√	√	√	√	?	?	√	Petri and Stengl, 1997; Werckenthin et al., 2012, 2020
*Tribolium castaneum*	√	x	√	√	√	√	√	√	√	√	?	√	Yuan et al., 2007; Ingram et al., 2012; Li C-J et al., 2018; Veenstra, 2019
*Apis mellifera*	√	x	√	x	√	√	√	√	√	√	√	√	Bloch et al., 2003; Rubin et al., 2006; Sumiyoshi et al., 2011; Beer et al., 2018

*Insect models of different orders and their clock genes. PER (PERIOD), CRY (CRYPTOCHROME) 1 and 2, TIM1 (TIMELESS) and TIM2 (TIMEOUT), CLK (CLOCK), CYC (CYCLE), PDP1 (PAR Domain Protein 1), VRI (VRILLE), CWO (CLOCKWORK ORANGE), JET (JETLAG), and the neuropeptide PDF (Pigment Dispersing Factor). Genes encoding these clock components are marked as present (√), absent (x) in genome or unknown (?)*.

* *PDF is found in other Hemiptera (Sato et al., 2002; Závodská et al., 2003)*.

The clock genes *per* and *tim1*/*cry2* and their respective protein products participate in a first negative feedback loop, in which the proteins inhibit the transcription of their own genes. This involves two further clock genes, *cycle* (*cyc*), and *Clock* (*Clk*), and their respective products CYC and CLK. CLK and CYC form heterodimers and bind to E-box regulatory elements in the promoters of *per* and *tim1/cry2*, activating their transcription. Consequently, *per* and *tim1/cry2* mRNA levels rise and are translated in the cytoplasm, where their products PER and TIM1/CRY2 are subjected to posttranslational modification, dimerize, and after a while enter the nucleus as a complex. In the nucleus, PER-TIM1 or PER-CRY2 complexes bind to CLK-CYC and repress their transcriptional activity. Doing so, they negatively regulate their own expression with a time delay. This delay is provoked by the posttranslational modifications of PER and TIM1/CRY2 and it is essential for provoking stable circadian oscillations. Subsequent PER and TIM1/CRY2 destabilization and degradation stops the repression on CLK-CYC activity, and a new transcriptional-translational cycle restarts.

The basic negative feedback mechanism is very similar in all animals (although gene sets differ), but again there are unique features in higher flies. While CYC (also called BMAL1 in mammals) is the component that binds to the E-boxes and activates transcription of *per* and *tim1/cry2* in the great majority of animals (including bees), CLK is the relevant transcriptional activator in higher flies (Bae et al., 1998; Chang et al., 2003; Rubin et al., 2006; Yuan et al., 2007; Sandrelli et al., 2008; Tomioka and Matsumoto, 2015; Chahad-Ehlers, 2017) (see Figure 1). Most interestingly, the transcription of the clock factor that possesses the transactivation domain is controlled in a rhythmic manner through a second feedback loop, while the one without transactivation domain is not rhythmically controlled. However, cyclic *Clk* expression has been observed for example in sandflies and in jewel wasps and crickets under certain conditions, although *cyc* encodes the transactivation domain (Meireles-Filho and Kyriacou, 2013; Uryu et al., 2013; Dalla Benetta et al., 2019) (see below).

## The Molecular Clock—Further Feedback Loops

There is a second feedback loop that leads to a circadian oscillation in the abundance of CLK in flies and of CYC (BMAL1) in the other animals (Cyran et al., 2003; Meireles-Filho et al., 2006a; Rubin et al., 2006; Sandrelli et al., 2008; Bertolini et al., 2018). This second feedback loop is so far best described in *D. melanogaster*. It involves the clock genes, *cycle* (*cyc*), *Clock* (*Clk*), *vrille* (*vri*), and *PAR domain protein 1* (*pdp1*), and their respective products. *Vri* and *pdp1* carry E-box regulatory elements in their promoters, therefore their expression is also activated by the active CLK-CYC complex. VRI accumulates earlier than PDP1 and it represses the expression of *Clk*, acting at the level of VP-boxes (Emery and Reppert, 2004) present in its promoter region. PDP1 accumulates later than VRI and finally promotes *Clk* expression. The synergistic activity of VRI and PDP1 generates circadian transcription of *clk*. However, different functions of the genes *pdp1* and *vri* of this second feedback loop are still largely undiscovered, because null-mutation studies are limited by the fact that the null-mutants exhibit developmental lethality. Besides their role in development of the fly, *pdp1* and *vri* were proposed to regulate output function of the clock downstream of the central oscillator, because changes in levels of PDP1 and the loss of *vri* in clock cells (*tim*- and *pdf*-expressing cells) caused arrhythmic behavior but did not affect core clock function (Benito et al., 2007; Gunawardhana and Hardin, 2017). The inhibition of the activity of a specific isoform of PDP1 (PDP1ε) and missing *vri* expression in clock cells furthermore displayed a role in regulation of clock neuron morphology and neuropeptide accumulation (Lim et al., 2007a; Gunawardhana and Hardin, 2017). In contrast, Zheng et al. (2009) confirmed *Clk* activation by PDP1ε and suggested that *pdp1* functions in both, core clock and behavioral output pathways.

As already mentioned, in Hymenoptera, *cyc* and not *Clk* appears to be rhythmically controlled, but exact mechanisms remain to be elucidated (Rubin et al., 2006; Ingram et al., 2012; Sadd et al., 2015). For example, in the jewel wasp, *Nasonia vitripennis*, a rhythmic control of *Clk* expression was found additionally to that of *cyc* in one study (Dalla Benetta et al., 2019) and only in *cyc* in another study (Davies and Tauber, 2016). Since *Clk* expression was only cycling in a *Nasonia* strain from Northern regions and only under long day conditions, but cycling was lacking in Southern species, Dalla Benetta et al. (2019) concluded that this may be due to an adaptation mechanism in the clock to photoperiods at higher latitudes. Overall expression levels of Clk and cyc were lower in northern *Nasonia* species (Dalla Benetta et al., 2019). Interestingly, there are parallels to the *cyc* knock-down studies in crickets, which also showed cycling in *Clk* expression when *cyc* levels are diminished (Uryu et al., 2013). This may point to a general mechanism of the circadian clock that promotes rhythmic *Clk* expression in case of low CYC levels.

With *cry1* and *tim1* lacking in the Hymenoptera clock, it may be that another transcription regulator, clockwork *orange* (*cwo*), plays a rather important role for core clock function, which is not yet understood (Ingram et al., 2012; Rodriguez-Zas et al., 2012). In *Drosophila, cwo* participates in a third feedback loop that influences CLK-CYC mediated transcription and regulates the amplitude of circadian oscillations in other clock genes (Kadener et al., 2007; Lim et al., 2007b; Matsumoto et al., 2007; Richier et al., 2008). The CWO protein promotes the PER-dependent removal of CLK-CYC complexes from E-boxes, which may be achieved by a binding competition between CWO and CLK-CYC-PER on E-boxes (Zhou et al., 2016). A CWO protein domain that is highly conserved amongst insects but different in the mammalian CWO orthologs DEC1 and DEC2 indicates that *cwo* function may be similar in all insect clocks (Ingram et al., 2012).

There may be more clock regulation factors in the fruit fly, which are still unknown. For example, only recently, a function in circadian clock regulation was postulated for the nuclear receptors ecdysone induced protein 75 (E75) and Unfulfilled (UNF), which may be conserved among different animals (Kumar et al., 2014; Jaumouillé et al., 2015).

The great advantage of *D. melanogaster* as a model in chronobiology is its well-described molecular mechanisms of the circadian clock and these mechanisms start to emerge also in other Diptera (Codd et al., 2007; Gentile et al., 2009; Rund et al., 2011; Meireles-Filho and Kyriacou, 2013; Kyriacou, 2014a; Gesto et al., 2015; Meuti et al., 2015; Kaiser et al., 2016; Bazalova and Dolezel, 2017; Bertolini et al., 2018; Noreen et al., 2018; Rivas et al., 2018). On the other hand, it is not possible to transfer all clock functions to other insect models as we illustrated above. Therefore, studies on various different insect clocks are needed to elucidate the role of the clock in complex behaviors, as we find it in other insects such as Hymenopteran species. In the following section, we will focus on the neuronal network of *D. melanogaster* and *A. mellifera* and show basic similarities and differences in these insect clock networks.

## The Circadian Clock Network of Fruit Flies and Honey Bees

**Figure 2:**
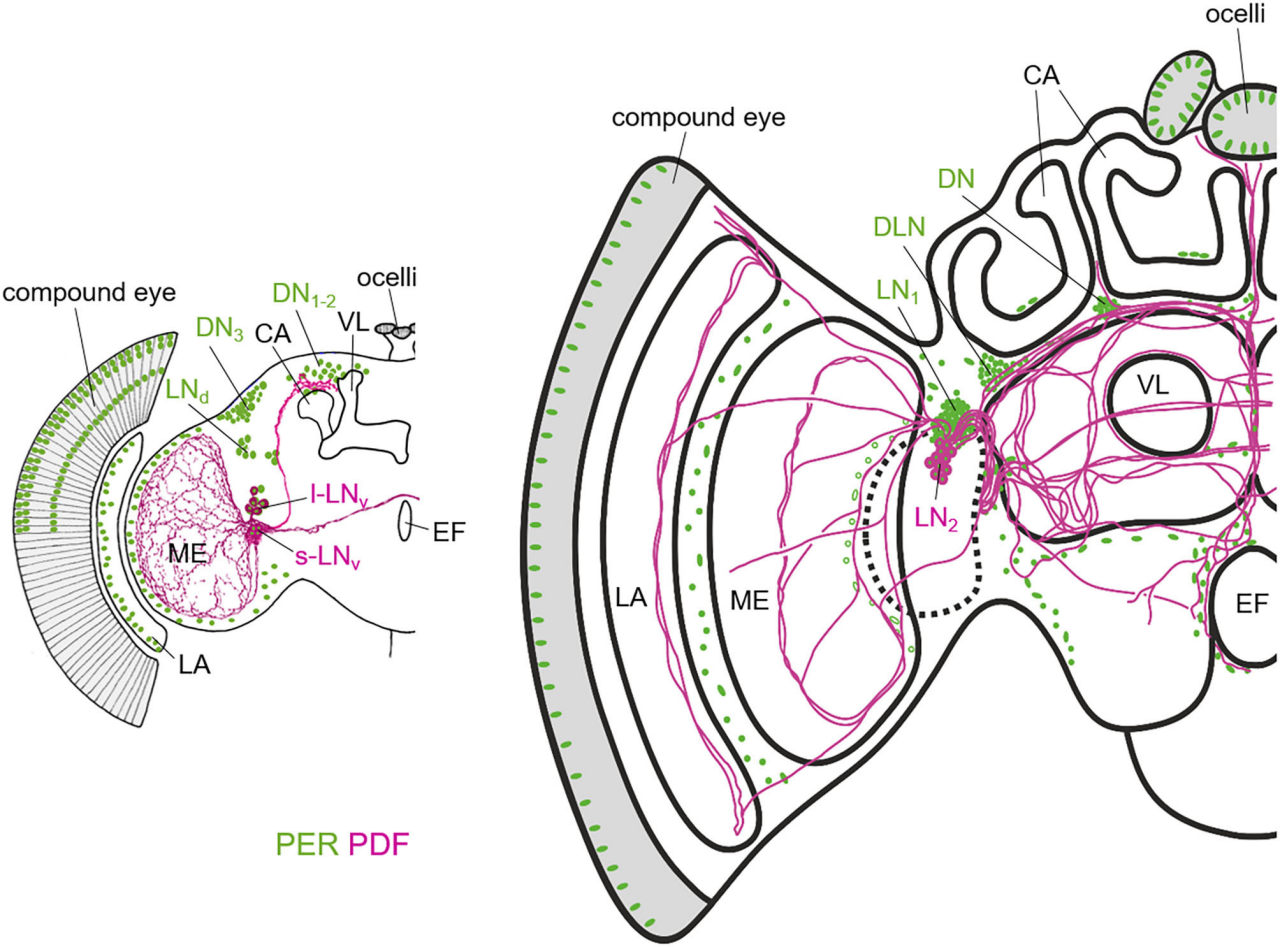
Schematic representation of the PER and PDF expressing neurons in the fruit fly **(left)** and honey bee brain **(right)**. The two brains differ in overall size, but especially in the size of the mushroom bodies of which the Calyces (CA) and the vertical lobes (VL) are highlighted. The vertical lobe is also called α-lobe in *Drosophila*. While the honey bee has two large calyces per brain hemisphere, the fruit fly has only one rather small one. In *Drosophila*, the only PDF-fibers that run into the dorsal brain, terminate anterior, and slightly dorsally of the CA. In the honey bee, the PDF fibers terminate anterior and ventrally of the CAs and they run into many more central brain areas than in *Drosophila*. PER is not only present in the lateral (LN) and dorsal neurons, but also in the photoreceptor cells of the compound eyes and ocelli and in numerous glia cells that are only indicated in the lamina (LA) and the distal medulla (ME) in *Drosophila* and in few other brain regions in the honey bee. Note that the nuclei of the photoreceptor cells are only shown in the dorsal half of the compound eye in *Drosophila*. The honey bee scheme is modified from Beer et al. (2018). For details see text.

In *D. melanogaster*, the central clock is located in dorsal and lateral neurons that express the core clock genes and form an extensive neuropeptidergic network in the brain (Figure 2). In addition, the clock ticks in many glia cells (Zerr et al., 1990). The clock neurons are traditionally divided into seven groups–three dorsal ones (DN_1−3_) in the dorsal brain, three lateral ones (LN_d_, l-LN_v_, and s-LN_v_) in the anterior lateral brain, and one additional lateral group in the posterior brain that is called LPN [reviewed in Helfrich-Förster et al. (2007a), Hermann-Luibl and Helfrich-Förster (2015), Helfrich-Förster (2017), Schubert et al. (2018)]. The clock neurons have two main projection targets: (1) the accessory medulla (aMe), a small neuropil situated between the central brain and the optic lobes and that had been identified as pacemaker center in many insect species and (2) the dorsal brain that houses the hormonal center (pars intercerebralis (PI) of insects and also has connections to most brain areas. The clock neurons form a well-defined fiber network in these two brain areas, putatively allowing considerable crosstalk between them. The aMe is not only invaded by the clock neurons but also by aminergic, glutaminergic, acetylcholinergic, and Glycin- and GABA-ergic inputs from non-clock neurons [reviewed in Helfrich-Förster (2017), Top and Young (2018)]. This emphasizes the role of the aMe in intercellular communication—both among clock neurons and between extrinsic cells and clock neurons. Furthermore, the aMe appears to get light information from the eyes and from the Hofbauer-Buchner eyelets (H-B eyelets), small extra retinal eyelets that are located beneath the compound eyes and are remnants of the larval stemmata [reviewed in Helfrich-Förster (2020)]. The aMe can be regarded as a hub to channel retinal and extra retinal inputs to the central circadian clock entraining it to the periodic environmental cycles (Li M-T et al., 2018). In the dorsal brain, the clock neurons' fibers terminate close to regions that have been shown to be involved in the control of locomotion, sleep, and metabolism, such as the PI, the mushroom bodies, and the central complex (see below under “***Behavior controlled by the circadian clock of fruit flies and honey*** ***bees”***).

One of the best conserved and most important neuropeptides in the insect circadian clock is the Pigment-Dispersing Factor (PDF) (Renn et al., 1999; Helfrich-Förster et al., 2000; Helfrich-Förster, 2014; Ikeno et al., 2014; Shafer and Yao, 2014; Wei et al., 2014; Beer et al., 2018). In *D. melanogaster*, PDF is expressed in four small ventro-lateral neurons (s-LN_v_) and in four large ventro-lateral neurons (l-LN_v_), which have different roles in the clock network. While the l-LN_v_ are dispensable for rhythmic activity, they signal to the s-LN_v_ (Klose et al., 2016; Schlichting et al., 2016; Menegazzi et al., 2017) and they are part of the light-input pathway to the clock (Helfrich-Förster, 2020).

The s-LN_v_ are major pacemaker neurons that are essential for robust rhythmic activity under constant darkness. Although the s-LN_v_ projections that terminate in the dorsolateral protocerebrum anteriorly of the mushroom body calyces look relatively simple, they appear to signal to different neuropils and downstream neurons via paracrine secretion of PDF. The s-LN_v_ terminals broaden and PDF accumulates in them in the morning (Park et al., 2000; Fernández et al., 2008) suggesting that it is also released at this time into the dorsolateral brain. PDF-receptors are for example on the ellipsoid body of the central complex (Pírez et al., 2013), which is a higher coordination center in the insect brain responsible for motor control and orientation and recently the ellipsoid body has been established as important output circuit downstream of the circadian clock neurons (Liang et al., 2019) (see also below).

However, PDF is not only a putative output factor of the clock but also the most important communication factor within the clock (Lin et al., 2004; Yoshii et al., 2009a; Helfrich-Förster, 2014; Shafer and Yao, 2014). The PDF receptor is expressed on many clock neurons, including the PDF-positive s-LN_v_ (Shafer et al., 2008; Im and Taghert, 2010; Choi et al., 2012; Klose et al., 2016). PDF is able to couple the molecular oscillations of individual clock neurons by speeding them up or slowing them down (Lin et al., 2004; Yoshii et al., 2009a). Thus, PDF is the most powerful neuropeptide in the clock network, leading to the hypothesis that the PDF neurons are dominant circadian pacemakers governing the other clock neurons by setting phase and period of their molecular clocks and shaping the activity pattern of the flies (Stoleru et al., 2005; Guo et al., 2014; Chatterjee et al., 2018).

In comparison to the fly, the honey bee brain possesses many more clock neurons (~400 compared to 150 in the fly brain), which are nevertheless clustered similarly like in the fly brain (Fuchikawa et al., 2017; Beer et al., 2018) (Figure 2). There are two rather dorsally located clusters, the DN (dorsal neurons) and the DLN (dorsolateral neurons), and two clusters (LN_1_, LN_2_) in the lateral brain between the protocerebrum and the optic lobe. The cell bodies of the LN_2_ neurons (~15 per hemisphere) are very closely located to the LN_1_, but are a little bit bigger and produce PDF (Fuchikawa et al., 2017; Beer et al., 2018). Like in *Drosophila*, the PDF neurons seem to take on a communication function between different clock cells and brain regions for control of downstream behavior, because injections of artificial PDF peptide have been successful to shift the locomotion rhythms in bees (Beer et al., 2018). The PDF neurons build a highly complex network of arborizations widespread throughout the honey bee brain. Like in other Hymenoptera, classification of PDF neurons in the honey bee into different functional groups, has not been possible so far (Bloch et al., 2003; Weiss et al., 2009; Sumiyoshi et al., 2011; Fuchikawa et al., 2017; Beer et al., 2018; Kay et al., 2018). Nevertheless, a functional subdivision of neurons projecting into different brain areas, like it is the case in *Drosophila* or cockroaches (Reischig et al., 2004; Helfrich-Förster et al., 2007b), is highly likely. Similar to other insects, the PDF neurons in the honey bee brain project into an highly dense fiber hub in the lateral brain close to the optic lobe (Homberg et al., 1991; Helfrich-Förster et al., 1998; Závodská et al., 2003). This “communication center” of the circadian clock may be analog to the aMe in *Drosophila* and other insects, with a small difference in location: it seems rather less associated with the Medulla than with the Lobula (Beer et al., 2018). This was suggested to be related to the fact that Hymenoptera have no stemmata, which are the precursor of the HB-eyelet in *Drosophila* development, and the developing honey bee clock may be consequently less associated with photic inputs (Beer et al., 2018).

Additionally to the clock neurons, numerous glia cells expressing PER (which were similarly observed in *Drosophila* (Siwicki et al., 1988; Helfrich-Förster, 1995) in various brain areas are closely connected via the PDF neuronal network (Fuchikawa et al., 2017; Beer et al., 2018). This fact and evidence from different *per* expression studies in nursing and foraging bees indicates a crucial role of glia cells in the circadian plasticity of the honey bee clock as we will explain later [see section “***Behavior*** ***controlled by the circadian clock of fruit flies and honey bees***” and review (Beer and Bloch, 2020)].

## The Relevance of Zeitgebers Differs Between Flies and Bees

Circadian clocks have to be synchronized to the 24-h day of the earth by Zeitgebers. The most reliable Zeitgebers are the daily light-dark (LD) and temperature cycles, but also social interactions, periodic vibration signals and the availability of food can serve as Zeitgeber. For most adult insects, light is the most important Zeitgeber, which is followed by temperature and social interactions, while the impact of food is only studied in some insects (see below).

The effectiveness of Zeitgebers is very different for developing insects that receive no light input at all, because they nest in cavities or mature underground, such as onion flies (Watari and Tanaka, 2010; Miyazaki et al., 2016), tsetse flies (Ždárek and Denlinger, 1995) and solitary bees (Tweedy and Stephen, 1970; Yocum et al., 2016; Bennett et al., 2018; Beer et al., 2019). For these insects the daily temperature cycle is the most important Zeitgeber for emerging rhythmically from their pupal case and at least solitary bees do not entrain to LD cycles when they are present (Tweedy and Stephen, 1970; Beer et al., 2019). Even fruit flies, which can perceive light through their pupal case and which are nicely entrainable by LD cycles in the lab are very sensitive to temperature cycles (Zimmerman et al., 1968), and under natural conditions, the daily phase of eclosion and the robustness of rhythmicity depends strongly on the environmental temperature conditions (Ruf et al., 2019). This indicates that temperature cycles may be generally more important for entraining the endogenous clock of developing insects than they are for entraining the clock of adult insects. During development the sensitivity to light-dark cycles appears to increase (Watari, 2005; Beer et al., 2019). In some flies, this switch to light sensitivity of the clock may occur earlier than in onion flies or solitary bees [see discussion in Beer et al. (2019)].

Furthermore, although adult insects are all very sensitive to LD cycles, for social insects, such as honey bees, social Zeitgebers can be more important than light (Beer et al., 2016; Fuchikawa et al., 2016). Thereby, different members of a colony can serve as social Zeitgebers: a group of foraging/forager-aged bees or even the single queen was shown to determine the colony rhythm in honey bees (Moritz and Sakofski, 1991; Frisch and Koeniger, 1994; Beer et al., 2016; Fuchikawa et al., 2016). Laboratory experiments with small groups of worker bees showed that the relative number of bees is important for social synchronization and that individual bees preferably adapt the rhythm of larger groups (Moritz and Kryger, 1994). The cues by which social synchronization is mediated between individual honey bees is so far unknown. It appears that pheromones, micro-climate and vibration signals could play a role, while direct tactile and visual contact between con-specifics could be excluded (Eban-Rothschild et al., 2012; Beer et al., 2016; Fuchikawa et al., 2016). Similar factors appear to influence the rhythms of flies. Although, flies are not classified as social, they form groups, interact with each other, adjusting their interactive behavior to group size (Rooke et al., 2020) and their clocks can be entrained by pheromones (Levine et al., 2002; Krupp et al., 2008) and vibrations (Simoni et al., 2014). Clearly, in flies, social synchronization has not the same significance as it has in bees, but studying it might help to unravel the underlying mechanisms.

**Figure 3:**
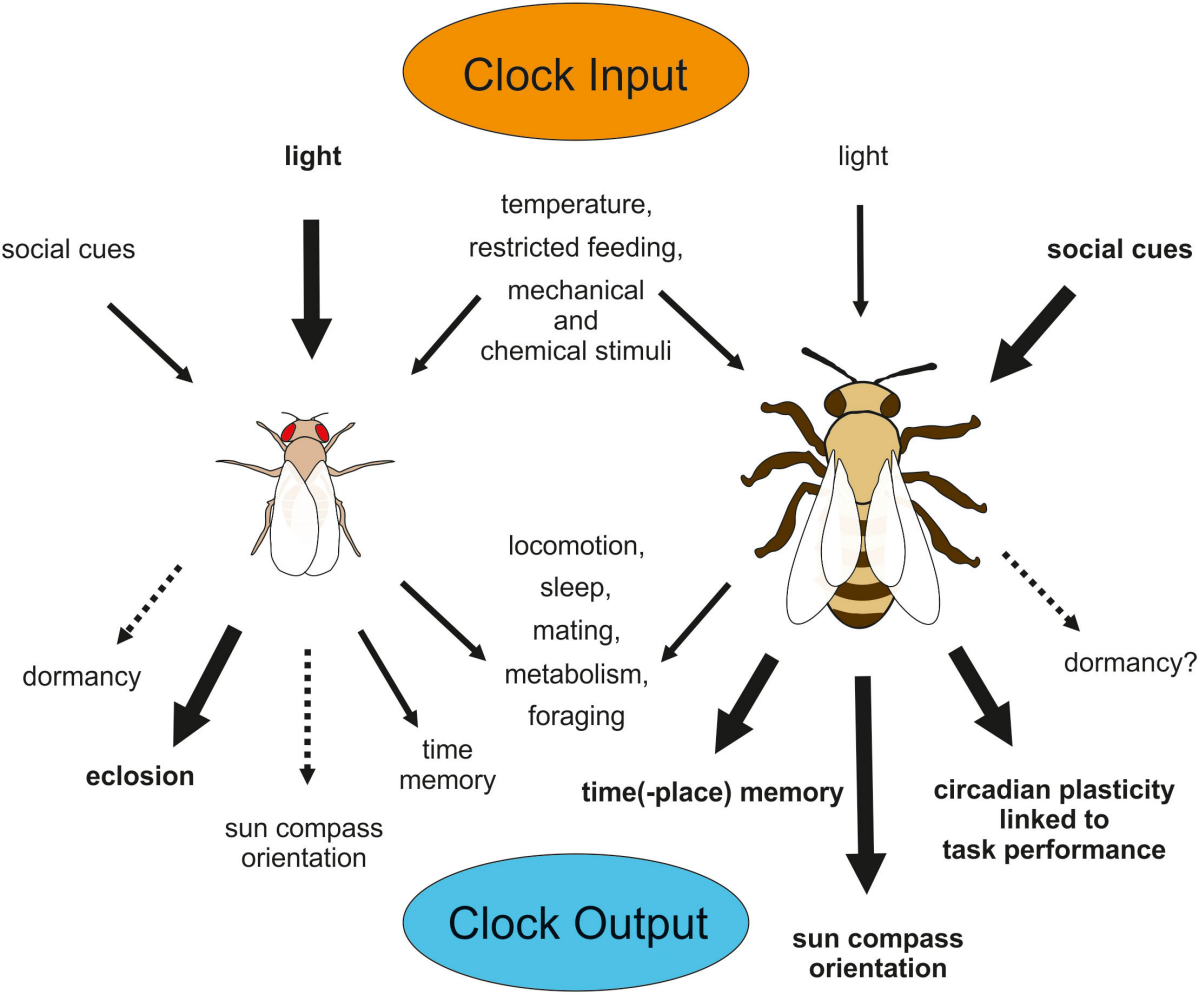
Input and Output of the circadian clock in *D. melanogaster* **(left)** and *A. mellifera* **(right)**. Relevance of input signals to the clocks differs between species, so that light appears to be most important for the fly clock, but social signals out rule light signals in entraining the bee clock. Many output behaviors are common among different insects (e.g., locomotion, sleep, mating, metabolism, and foraging), but others are limited to or more pronounced in some species (e.g., eclosion in *Drosophila* and time-place-memory, sun compass orientation or task-related plasticity in circadian behavior in *Apis*). Although *D. melanogaster* does not exhibit pronounced diapause, basic mechanisms in clock regulation involved in dormancy appear to be shared between this fly and diapausing fly species. Similarly, *A. mellifera* does not go into diapause, but it may be that the honey bee exhibits some residual photoperiodic function in initiating dormancy behavior, because solitary bees display pronounced diapause.

In the following, we will give an overview about the effectiveness of light and temperature cycles as Zeitgebers in adult flies and bees (Figure 3). For a detailed insight into light-input pathways to the circadian clock of adult insects with an emphasis on the fruit fly the reader is referred to a recent review (Helfrich-Förster, 2020).

Adult fruit flies entrain to LD and temperature cycles [reviewed in Helfrich-Förster (2017)]. When both Zeitgebers are combined in a natural-like way with the highest temperature occurring after noon, locomotion rhythms are very precise and the molecular clock cycles with high amplitude in all clock neurons (Currie et al., 2009; Yoshii et al., 2009b). When LD and temperature cycles are completely out of phase with each other, wild-type flies entrain to the LD cycles, while mutants without CRY1 entrain to the temperature cycles, suggesting that light is the predominant Zeitgeber and light entrainment is mediated via CRY1 (Yoshii et al., 2010). However, this conclusion is slightly premature. When the two Zeitgeber cycles are <4 h out of phase, the temperature cycles strongly influence the phase of the activity rhythms, meaning that light and temperature interact in the entrainment of wild-type flies and that light only dominates when the two Zeitgebers are too much out of phase (Harper et al., 2016). That the compound eyes and not only CRY1 contribute to light entrainment under conflicting Zeitgeber cycles was shown by Busza et al. (2007). The compound eyes together with CRY1 actively suppress temperature input to the clock neurons in wild-type flies and by this way make sure that the flies are not too sensitive to sudden temperature fluctuations that can easily happen in nature (Busza et al., 2007; Gentile et al., 2013). Most interestingly, different clock neurons are responsible for mediating light- and temperature entrainment in fruit flies: the Dorsal Neurons are more important for temperature entrainment than the Lateral Neurons, which mediate predominantly light entrainment [reviewed in Helfrich-Förster (2017), Yadlapalli et al. (2018), Lamaze and Stanewsky (2020)].

The light-input pathway to the circadian clock in honey bees is less well-studied. Honey bees miss in comparison to the fruit fly the blue light receptor CRY1 and the HB-eyelet. Similar to CRY1-missing *Drosophila* mutants, the honey bee clock may be less susceptible to light than other environmental cues (and at least for social cues this theory seems to hold true). With *tim1* absent in the molecular clock of honey bees, the light input pathway in bees may utilize another mechanism. *Tim2* (*timeout*) was identified as part of a photo-entrainment mechanism in *Drosophila* (Benna et al., 2010), which only plays an residual role in photoreception compared to *tim1* in *Drosophila*. The relevance of the mammalian ortholog of *tim2* for the circadian system is highly debated until today (Gotter et al., 2000; Barnes, 2003; Gotter, 2006). However, with *cry1* missing, a *tim2* mediated photoreception in the compound eyes may be the major photo-entrainment pathway in Hymenoptera (Benna et al., 2010). Furthermore, a vertebrate-like opsin called pteropsin, may be part of the light-input pathway to the clock in honey bees besides the compound eyes and the ocelli (Velarde et al., 2005). The expression pattern of pteropsin strongly resembles the location of PER expressing clock cells (Fuchikawa et al., 2017; Beer et al., 2018).

## Behavior Controlled by the Circadian Clock of Fruit Flies and Honey Bees

Basic circadian clock output behavior is similar between fruit flies and honey bees. However, honey bees have an extraordinary rich behavioral repertoire and, due to their age- and caste-dependent differences in behavior, they are perfect models to study circadian clock development and plasticity as well as socially regulated clock output. Therefore, we will first review the general rhythmic behavior of flies and bees and then concentrate on honey bee behavior.

## Output Rhythms in Flies and Bees

The best studied daily rhythms in flies and bees are those of locomotion and of sleeping/waking [reviewed for flies by Dubowy and Sehgal (2017), King and Sehgal (2020), and for bees by Moore (2001), Eban-Rothschild and Bloch (2012)]. The honey bee has been found to be a very good model for sleep, because of its detailed description of sleep architecture (Kaiser, 1988; Sauer et al., 2003; Klein et al., 2014), while the fruit fly has been very helpful to unravel the underlying molecular and recently also neuronal mechanism of sleep [reviewed in Helfrich-Förster (2017), Guo et al. (2018)]. In both insects, disturbances of the sleep-wake-rhythm result in reduced learning ability (Hussaini et al., 2009; Toda et al., 2018; Donlea, 2019) and in honey bees additionally in reduced communication ability, which is very similar in humans (Klein et al., 2010), suggesting that sleep is essential to maintain neuronal plasticity, learning and memory in all animals.

The daily rhythm in movement (locomotion), which is best studied in all insects investigated so far, can serve different purposes. Insects may be active for foraging, for seeking mates, for nesting/brood care activity or just because their circadian clock dictates them to be active. In most laboratory systems that record movements of insects, it is impossible to distinguish between these different possibilities. Here, natural studies with honey bee foragers are of great advantage. Indeed, their foraging rhythms are the first behavioral rhythms described in honey bees (Beling, 1929; Wahl, 1933; Kleber, 1935; Frisch and Aschoff, 1987; Moore et al., 1989). These studies showed that honey bees forage throughout the day depending on the available food sources and that they have an excellent memory about time and location of open flowers. When trained in restricted feeding cycles, honey bee foragers can remember up to nine time-points per day (Koltermann, 1971). Although, entrainment via feeding has been deployed in many studies, the link between the clock and time-place-learning in foraging behavior of bees is largely unknown (Pahl et al., 2007; Moore and Doherty, 2009; Mulder et al., 2013). Only recently, it was shown that restricted feeding indeed can phase shift the molecular clock of honey bees (Jain and Brockmann, 2018).

When recorded in isolation and under controlled conditions in the lab, honey bee foragers show activity throughout the day and few activity during the night (Moore and Rankin, 1985). Fruit flies have a completely different activity pattern under such conditions. They exhibit bimodal activity rhythms with activity bouts in the morning and evening and a pronounced siesta in between, and this is true for both sexes (Helfrich-Förster, 2000). Nevertheless, there are differences between the sexes. Mated females show a greatly reduced siesta, probably because they search for places for depositing their eggs. Oviposition occurs rhythmically in female flies starting in the middle of the day and reaching a maximum in the evening (McCabe and Birley, 1998; Sheeba et al., 2001; Manjunatha et al., 2008), which fits to the high activity of isolated mated females during this time. In contrast, isolated males begin activity significantly earlier in the morning than females, which can be explained by a search for female mating partners (Helfrich-Förster, 2000). Indeed, male sex drive behavior has been shown to be controlled by the circadian clock (Fujii et al., 2007, 2017; Fujii and Amrein, 2010) and mating occurs rhythmically with a maximum in the early morning hours (Sakai and Ishida, 2001; Lin et al., 2014). Nevertheless, sex drive is influenced by the presence of females and is not generally restricted to the morning: male flies that are housed together with females become highly active throughout the night and early morning (Fujii et al., 2007). Similar to activity, feeding occurs at slightly different times of the day in male and female flies. While males feed maximally in the early morning, females do so from the middle of the day until the evening (Seay and Thummel, 2011; Xu et al., 2011; Liu et al., 2020; Schäbler et al., 2020). In summary, general activity, mating and feeding occur at different times of the day and additionally show sex differences in timing. Thus, these three rhythms may be controlled by different neuronal pathways from the clock neurons to the effector organs.

Unlike in the honey bee, no studies under real natural conditions have been performed in fruit flies and only few studies have addressed fly behavior under quasi natural conditions (Vanin et al., 2012; Green et al., 2015). Thus, we don't know yet, which activities are performed by fruit flies in nature and when they occur naturally. Nevertheless, many more rhythms are known from fruit flies studied under laboratory conditions, of which some have been also found in honey bees, while others appear absent in honey bees. For example, fruit flies do not only lay their eggs in a rhythmical manner, but they also eclose rhythmically from their pupal case, with a peak of eclosion in the subjective morning (Pittendrigh, 1954; Lin et al., 2014). Similar rhythms in the queen's oviposition or rhythms in emergence of newly eclosed honey bees could not be detected in honey bees (Free et al., 1992; Harano et al., 2007; Johnson et al., 2010). Most probably, such rhythms have no selective advantage in the protected beehive (but they do so in solitary bees, see above). Nevertheless, mating between drones and queens happens in a rhythmic manner as it does in fruit flies. Mating of bees always occurs in the afternoon (Lensky and Demter, 1985), while the exact timing of mating flights can be altered by selective forces (e.g., the presence of sympatric species within the same location). Generally, honey bees strongly avoid an overlap in flight times between sympatric species, either to avoid interspecific hybrids or a reduction in the efficiency of mating (Koeniger and Koeniger, 2000). We expect that such selective forces will also alter the timing of the different rhythms in fruit flies under natural conditions.

As do bees, flies can also remember the time of day, at least to some degree (Chouhan et al., 2015). Chouhan et al. (2015) showed that flies can remember two time points per day, as long as the two are at least 6 h apart. This memory depended on a functional circadian clock and did neither persist in the absence of the PER protein nor in the absence of the neuropeptide PDF. Time memory in honey bees is much more sophisticated than in flies, but it may relay on the same connections of the circadian clock neurons with the centers of memory, the mushroom bodies (see below). Furthermore, in flies and bees, the ability to learn is modulated by the circadian clock and different at different times of day (Sakai et al., 2004; Lehmann et al., 2011). Again, this requires a functional relationship between the clock neurons and the mushroom bodies.

## Sun Compass Orientation

Honey bees and other insects are famous for their remarkable spatial orientation, which relies on a time-compensated sun-compass (Lindauer, 1960). Because of the earth's rotation the relative position of the sun changes during the day and the bee has to compensate for the past time during flight. In several studies, in which the honey bee clock was phase shifted, it was shown that the circadian clock is essential for sun-compass orientation (Medugorac and Lindauer, 1967; Cheeseman et al., 2012, Cheeseman et al., 2014). Recently, putative input neurons of the clock to the sky compass orientation pathway in the honey bee brain have been identified (Zeller et al., 2015; Beer et al., 2018). Transmedulla neurons of the sky compass pathway originating at the dorsal rim area of the medulla run in close proximity to PDF neurons (Zeller et al., 2015). PDF neurons, on the other hand may communicate with the central complex, which generally controls orientation and navigation in insects (e.g., Pegel et al., 2019).

Also flies can fly straight over long distances, indicating that they can perceive celestial cues and might even be able to compensate for the movements of the sun although this is not yet proven (Giraldo et al., 2018; Mathejczyk and Wernet, 2019; Warren et al., 2019). If true, research on flies may help to decipher the neuronal pathway from polarization vision to the circadian clock and from there to the brain centers controlling orientation such as the central complex. In any case, good interdisciplinary communication between fly and bee researchers will strongly support the successful elucidation of sun-compass orientation mechanisms.

## Task Related Plasticity of the Clock in Honey Bees

Honey bees have an age-related division of labor displayed by worker bees. At the age of 2–10 days bees assume brood care (nursing) functions in the colony, later they take over other duties such as storing and processing food or guarding the hive and around 3 weeks of age they begin to forage pollen or nectar and are now called foragers (Free, 1965). This division of labor is associated with pronounced changes in rhythmic behavior (Crailsheim et al., 1996; Moore et al., 1998; Bloch and Robinson, 2001). Typically, young bees tend the brood without a rhythm in locomotion, which is supposed to be beneficial for optimizing brood care and colony growth. They also display more and less pronounced sleep-bouts scattered over the day (Eban-Rothschild and Bloch, 2008; Klein et al., 2008). Older foraging bees on the other hand, display robust day-night rhythms of activity and sleep. This behavior is highly plastic and bees can start prematurely rhythmic foraging or revert back to nursing without rhythms, all according to the need of the colony (Bloch and Robinson, 2001). The cues establishing arrhythmic behavior in bees are largely unknown, but contact to the brood is essential (Nagari and Bloch, 2012; Nagari et al., 2017a). The plasticity in this behavior is further demonstrated by the fact that nurse bees displayed rhythmic behavior (locomotion) shortly after removing them from the colony (Shemesh et al., 2007, 2010; Eban-Rothschild et al., 2012; Fuchikawa et al., 2016; Nagari et al., 2017b). Moreover, molecular studies were in line with the behavioral experiments and showed attenuation of the circadian rhythm in mRNA levels of the clock genes *per, cry2, cyc*, and *cwo* in nurses compared to foragers (Bloch et al., 2001; Shemesh et al., 2007, Shemesh et al., 2010; Rodriguez-Zas et al., 2012). This implies a major reorganization of the circadian clock system. But does the clock of nurse bees actually stop? It was puzzling to see that the clock of nurses drove activity rhythms in the laboratory that were in phase with the ambient day-night rhythm of the colony, they had been removed from. Furthermore, PER protein levels were cycling in brains of behaviorally arrhythmic nurse bees (Fuchikawa et al., 2017). That means that the clock in nurse bees keeps ticking even when they are behaviorally arrhythmic! Still, why is it that mRNA oscillation of clock genes is attenuated in comparison to forager bees? As we have already mentioned, the numerous PER expressing glia cells may play an essential role in the honey bee clock. In *Drosophila* and mammals, it was demonstrated that non-neuronal cells are part of the circadian clock and contribute to formation of rhythmic behavior (Ng et al., 2011; Jackson et al., 2015; Brancaccio et al., 2017). Similarly, the PER expressing glia cells could be involved in circadian plasticity in honey bees. A differential coupling of clock cells in nurses and foragers may also be possible in the highly complex circadian clock network of honey bees. Future studies may help to elucidate the regulation mechanism of circadian plasticity in honey bees.

Interestingly, also queens display circadian plasticity in their behavior. In the colony, they were observed to be behaviorally arrhythmic while laying eggs (Free et al., 1992; Johnson et al., 2010). However, when isolated in the laboratory, they show rhythms in locomotion in phase with the ambient day-night cycle (Harano et al., 2007).

## Neuronal Output Pathways From the Clock to Downstream Neurons

Although circadian clock output behaviors have been studied in numerous insects, the output pathways from the circadian clock in the brain to rhythmic behavior have been revealed only lately and in many cases are not well-understood yet. Here, again *Drosophila* with its manifold genetic tools has been the pioneer model. In the following, we will briefly review the different possible neuronal pathways from the clock to downstream neurons in the brain that may in turn communicate with the body.

Starting with development and eclosion from the pupal case, Selcho et al. (2017) showed that the s-LN_v_ transfer timing information via the neuropeptide sNPF (small neuropeptide F), which is co-produced with PDF, to neurosecretory cells in the dorso-lateral brain that produce the neuropeptide PTTH (Prothoracotropic hormone). PTTH then forwards time information to the prothoracic gland, which secretes the steroid molting hormone, ecdysone. The titer of ecdysone gates subsequent eclosion. The mating rhythm of adult flies appears to be correlated with pheromone release from the oenocyctes and the latter is coupled to the circadian clock in the brain via PDF (Krupp et al., 2013). The precise pathways of this regulation are however not yet known.

Sleep is controlled by the central complex (besides other brain areas) and a connection from the clock neurons to the ellipsoid body of the central complex has been identified by Guo et al. (2018), Lamaze et al. (2018), and Lamaze et al. (2018) [nicely summarized by Hsu and Sehgal (2018)]. These authors show that specific DN_1_ clock neurons that get input from the s-LN_v_ contact so-called tubercular-bulbar (TuBu) neurons that in turn are connected to ellipsoid body ring neurons that promote sleep. Most importantly, these ellipsoid body ring neurons are different from those involved in spatial orientation (see above). They are also different from ring neurons that have been recently shown to be implicated in the control of the flies' bimodal activity in the morning and evening (Liang et al., 2019). Thus, there are several parallel pathways ending in specific neurons of the ellipsoid body. Liang et al. (2019) demonstrated that other specific ring neurons of the ellipsoid body display spontaneous morning and evening neural activity peaks that coincide with the bouts of locomotion and that get inputs from circadian clock neurons that control morning and evening activity, respectively. This input is indirect and occurs via specific dopaminergic neurons that also arborize in the ellipsoid body. The s-LN_v_ control morning activity and PDF is able to activate the dopaminergic neurons as well as the ellipsoid body ring neurons (Liang et al., 2019). Thus, PDF may be one of the clock factors that signals directly to the ellipsoid body [see Pírez et al. (2013) and above], while the connection from the evening clock neurons to the ellipsoid body neurons is still unknown [for more information on morning and evening clock neurons see Yoshii et al. (2012)]. Nevertheless, activity is controlled also by other parallel pathways that circumvent the ellipsoid body and run via the hormonal center, the PI in the middle dorsal brain [reviewed in King and Sehgal (2020)]. One important humoral pathway runs via six PI neurons that produce Diuretic Hormone 44 (DH44) a homolog of the mammalian stress hormone releasing factor (Cavanaugh et al., 2014). DH44 neurons receive synaptic inputs from the DN_1_ clock neurons and DH44 is important for strong activity rhythms under constant darkness. However DH44 neurons are most active during mid-day (Bai et al., 2018) making it likely that they promote activity for other reasons. Possibly, they elevate activity for searching food or egg-laying places in females. In addition to the DH44 neurons, there are further neurons in the dorsal brain that are contacted by the clock neurons and contribute to shaping activity (Pírez et al., 2019).

Feeding rhythms *per se* are controlled by a different set of PI neurons, the four SIFamide positive neurons (Dreyer et al., 2019). The SIFamide neurons are also contacted by the DN_1_ clock neurons and project to the subesophageal ganglion that is involved in gustatory processing and contains feeding-related motor neurons. Indeed, stimulation of the SIFamide neurons increases food intake (Martelli et al., 2017). Feeding is additionally controlled by leucokinin-positive neurons that are downstream of the s-LN_v_ and other clock neurons (Cavey et al., 2016) and that mediate hunger signals to promote locomotion (Zandawala et al., 2018; Yurgel et al., 2019). Finally, the PI contains 14 Insulin-like-Peptide positive neurons (IPCs) that are contacted by the DN_1_ and the s-LN_v_ clock neurons and control circadian gene expression in the fat body (Barber et al., 2016) and general metabolism (see next chapter).

**Figure 4:**
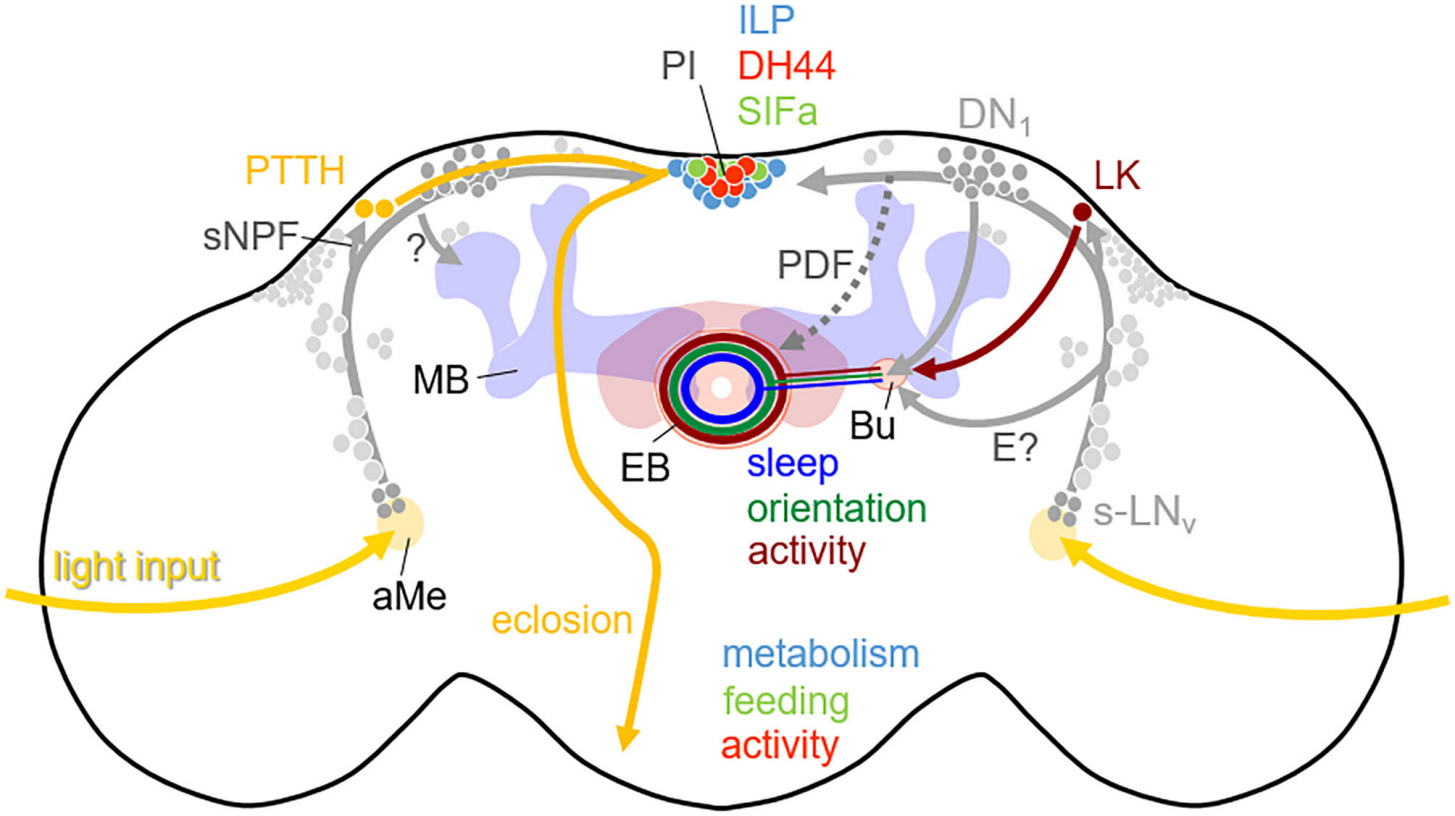
Simplified scheme showing the output pathways of the clock neurons in the brain of *D. melanogaster*. The somata of the clock neurons in the lateral and dorsal brain are depicted in gray with the s-LN_v_ and DN_1_ highlighted. The clock neurons are connected with each other and most of them have neurites in the accessory medulla (aMe), where they receive major light-input, as well as in the dorsal brain where they connect different neuropeptidergic neurons. The prothoracotrophic hormone (PTTH) positive neurons receive rhythmic signals via sNPF from the s-LN_v_ and control the timing of eclosion by triggering the release of ecdysone from the prothoracotropic glands (shown in the left brain hemisphere). The leukokine (LK) positive neurons receive also rhythmic signals from the s-LNv and perhaps from other lateral neurons and contact via tubercular-bulbar neurons (not shown) the bulb (Bu) where ring neurons of the ellipsoid body (EB) have their dendrites (shown in the right brain hemisphere). The corresponding EB ring neurons contribute to the rhythmic control of activity. In addition, the morning neurons (s-LN_v_) and evening neurons (not highlighted) signal via PDF (stippled) and unknown pathways (E?) to dopaminergic neurons (not shown) that arborize in the Bu and the EB and contact putatively the same activity promoting ring neurons in the EB in order to stimulate morning and evening activity. Other ring neurons in the EB receive rhythmic signals from the DN_1_ and control sleep, while still others may get rhythmic input from the clock neurons via still unknown pathways and control orientation. Furthermore, activity, feeding and metabolism are controlled by diuretic hormone 44 (DH44), SIFamide (SIFa), and Insulin-like peptide (ILP) expressing neurons in the pars intercerebralis (PI), which receive rhythmic signals from the clock neurons. Finally, the clock neurons control learning and contribute to time memory via still unknown pathways (?) to the mushroom bodies (MB).

In summary, there are multiple output pathways from the circadian clock neurons that all originate in the dorsal brain (reaching from lateral to mid-central brain areas) (Figure 4). In the honey bee, these output pathways have not been elucidated in detail, but one can easily see in the pattern of the PDF arborizations that these neurons alone can easily establish contacts with the relevant brain areas mentioned for *Drosophila* (Figure 2). It will be most interesting to reveal the arborizations of the other clock neurons of the honey bee, especially with regard to the integration of the honey bee complex behaviors in the clock output network.

## The Role of the Clock in Photoperiodism and Diapause Needs New Insect Models

A central question in chronobiology is how endogenous clocks changed in order to anticipate vastly different cyclical environmental conditions on earth, especially such that exist close to the poles. Organisms like *D. melanogaster*, and also *Homo sapiens*, are assumed to have developed in tropical regions that are characterized by regular 24 h cycles in irradiance and temperature that remain the same throughout the seasons. However, in northern and southern hemispheres of the earth photoperiods vary throughout the year causing the well-known seasons of spring, summer, autumn and winter. All organisms including insects have to anticipate these seasons in order to be prepared in advance for the coming spring-summer and autumn-winter. Bünning (1936) hypothesized that the main purpose of the circadian clock is to provide the necessary time reference for measuring day length so that organisms can prepare in time for the winter. A failure in such a preparation will ultimately lead to death. Similarly, a failure to predict the coming warm season will lead to the death of the offspring, since a too early or too late reproduction may result in too low temperatures and/or shortages of food. Small animals, such as insects are especially sensitive to seasonal changes and need to be well-prepared.

One strategy of avoiding adverse conditions is reproductive arrest that is also called dormancy or diapause. Dormancy is a generic term covering any state of developmental arrest, which is adaptive and usually accompanied with metabolic suppression (Koštál, 2006). As soon as the adverse environmental conditions disappear, insects can terminate dormancy. Diapause is a specific subtype of dormancy, which is a more profound, endogenously and centrally mediated interruption that routes the developmental program away from direct morphogenesis into an alternative diapause program of succession of several physiological events. The start of diapause usually precedes the advent of adverse conditions. Since temperature alone is not a reliable predictor, most organism use day length (= photoperiod) as a measure for the coming season and diapause is induced as soon as day length falls beyond a certain threshold, the critical day length (Koštál, 2006). The responses to changing photoperiods are called photoperiodic responses. This is different for the obligatory diapause, which is present in insects that complete only one generation each year (Denlinger et al., 2017). Typical examples are solitary bees or *Rhagoletis* fruit flies. Such insects do not need a mechanism to measure day length for diapause induction, because they enter diapause at a fixed developmental stage regardless of prevailing environmental cues. However, environmental cues remain essential for regulating the timing of diapause termination, because the mechanism for terminating diapause at the appropriate time dictates the active window of the insect's life.

Tropical insects are not exposed to seasonal differences in photoperiod and don't have to undergo winter diapause. Thus, they have no need to measure day length. This does not mean that they don't undergo dormancy or even diapause, just other cues such as temperature, moisture, and changes in food quality dictate the induction of dormancy (Denlinger, 1986). Our model organisms, the honey bee, *Apis mellifera*, and the fruit fly, *D. melanogaster*, stem from tropical regions. Honey bees live additionally in hives and can actively produce heat; both protect them from the coldness of winter. Therefore, both species do not exhibit a photoperiodically induced diapause. Nevertheless, they undergo a state of dormancy in response to low temperatures and shortage of available food that can be enhanced by shortening day length (Kefuss, 1978; Saunders et al., 1989). In contrast to real diapause this state does not include a succession of defined physiological events and it can be terminated at any time when the environmental conditions improve.

Can we, nevertheless, learn something about the mechanisms of dormancy from *D. melanogaster*? The adult female flies exhibit a reproductive dormancy manifested by reduced metabolic activity and arrested ovarian development, which is stimulated by low temperatures and can be enhanced by short natural photoperiods and food shortage (Nagy et al., 2018, 2019; Ojima et al., 2018). As in other insects, the insulin-like peptide (ILP) producing cells (IPCs) are key regulators of this process, since they produce and release insulin-like peptides that act as diapause-antagonizing hormones. Although, fruit flies have no photoperiodically induced diapause that needs communication with the endogenous clock to determine day length, it was recently shown that the circadian clock neurons communicate with the IPC cells (Nagy et al., 2019). The s-LN_v_ clock neurons activate the IPCs via the neuropeptides Pigment-Dispersing Factor (PDF) and short neuropeptide F (sNPF), which in turn release insulin-like peptides, antagonize dormancy and lead to reproductive growth. This result suggests that the secretion of PDF and sNPF is enhanced under long summer days and by this way keep the flies in the reproductive state. PDF is indeed secreted during the day (Park et al., 2000), but it is still unknown whether its secretion is prolonged or enhanced under long days.

That the clock communicates with the dormancy inducing centers in the central brain of *D. melanogaster* flies can also be inferred from the fact that some components of the molecular circadian clock affect dormancy incidence. For example, a long isoform of the clock protein TIM (L-TIM) evolved a few 100–1,000 years ago, after *D. melanogaster* colonized Europe (Sandrelli et al., 2007; Tauber et al., 2007; Zonato et al., 2018); reviewed in Kyriacou (2014b). This long isoform coexists with the original short form (S-TIM), and flies carrying both isoforms (“LS-TIM” flies) gradually invaded Northern Europe and North America (Pegoraro et al., 2017). LS-TIM has a reduced ability to interact with CRY, which makes the flies less light-sensitive and less likely to become arrhythmic under extreme long days. In addition, the “LS-TIM” flies enter dormancy earlier in autumn than the S-TIM flies. Both properties are advantageous for a life in the north. A very recent study supports the role of TIM in dormancy of *D. melanogaster* (Abrieux et al., 2020). The authors showed that *tim* null mutants exhibit reduced incidence of reproductive dormancy in simulated winter conditions, while flies overexpressing *tim* show an increased incidence of reproductive dormancy even under long photoperiods.

What about insects that exhibit a real photoperiodic diapause? Most interestingly, the *tim1* or *cry1* clock genes plays also a role in the photoperiodic response of such species as for example the Japanese fruit fly *Chymomyza costata* (Stehlík et al., 2008), the fruit fly *Drosophila triauraria* (Yamada and Yamamoto, 2011), the silkworm *Bombyx mori* (Li, 2011), the Asian Tiger mosquito *Aedes albopictus* (Huang et al., 2015), and the Northern house mosquito *Culex pipiens* (Meuti et al., 2015). In photoperiodic species that possess no TIM1 and no light-sensitive CRY1 such as the bean bug *Riptortus pedestris* or the Linden bug *Pyrrhocoris apterus* other clock genes such as *per* or *Clk* are involved in the photoperiodic response (Syrová et al., 2003; Ikeno et al., 2010, 2011a,b).

Analogous to *D. melanogaster*, a knockdown of the neuropeptide PDF caused female *Culex pipiens* that were reared under long day conditions to enter a diapause-like state (Meuti et al., 2015). Furthermore, the ablation of the PDF-positive clock neurons in the blow fly *Protophormia terraenovae* interferes with photoperiodic diapause induction in such a way that the flies could not discriminate long and short days and half of the flies entered diapause at both conditions (Shiga and Numata, 2009). This confirms the importance of PDF as signaling molecule from the circadian clock to the IPC cells.

In most cases, PDF signaling to the IPC cells appears to keep flies in the reproductive state. Most interestingly, several *Drosophila* species such as *D. montana, D. littoralis, D. ezoana*, and *D. virilis* that live in the very north lack PDF in the s-LN_v_ clock neurons that project to the IPC cells (Bahn et al., 2009; Kauranen et al., 2012; Hermann et al., 2013; Menegazzi et al., 2017; Beauchamp et al., 2018); reviewed in Helfrich-Förster et al. (2018). These species have a high incidence of reproductive arrest already under long-day lengths, which is an adaptation to the low temperatures even under summer photoperiods at these clines. For example, *D*. *ezoana* enters diapause when day-length falls below 16 h (Vaze and Helfrich-Förster, 2016). The lack of PDF-signaling to the IPCs of these species might facilitate the termination of the reproductive state already at these relatively long days. In addition to lacking PDF in the s-LN_v_ clock neurons, these high-altitude flies lack CRY in other clock neurons: the l-LN_v_ [reviewed in Helfrich-Förster et al. (2018)]. This may enhance the flies' ability to enter dormancy earlier in the seasons as true for the less light-sensitive TIM-LS flies of *D. melanogaster*.

Nevertheless, not all fly species that are adapted to high altitudes lack PDF and CRY in certain clock neurons. For example, *C. costata* flies that are distributed in Eastern Siberia, Northern Lapland, Iceland, and from northern Japan to the Artic Cycle (Hackman et al., 1970) possess a *D. melanogaster*-like PDF network (Bertolini et al., 2019). This shows that the circadian clock of *C. costata* flies has found other ways to adapt to high-latitudes. PDF may just promote metabolic and reproductive activity, but there is no prove that it is really necessary for photoperiodic information. On the contrary: most photoperiodic species lack PDF signals to the IPC cells and nevertheless undergo diapause at a critical day length. For example, in the aphid *Acyrthosipon pisum*, which is a classic model for photoperiodism, PDF was even not found at all (Beer, Colizzi and Helfrich-Förster, unpublished). Furthermore, we still lack a detailed pathway leading from photoreception to expression of diapause. Though a functional circadian clock appears essential for the diapause response, it is not at all clear how the circadian clock and the photoperiodic timer are integrated. How are short days distinguished from long days, and how is this critical information stored in the brain to be acted upon at a later stage or even in the following generation?

Although most insects enter dormancy at some point in their life cycle, insects that currently offer the best models for genetic research lack a robust photoperiodic diapause. Further development of genetic tools for non-model species, including both loss and gain-of-function mutations, are urgently needed to advance the exciting field of insect photoperiodism. In addition, laboratory-based experiments can benefit from carefully simulated natural environments under controlled conditions, and whenever possible experiments should also be carried out in the wild. The natural world offers an incredibly rich diversity of biological clocks that can be probed for understanding the timing of seasonal activity.

## Concluding Remarks on Evolution of the Circadian Clock in Insects

As we have elucidated above, the model insects *D. melanogaster* and *A. mellifera*, have remarkable advantages in representing different aspects in chronobiology: Their genomes are sequenced, and many components of the circadian clockwork are already identified. They show a variety of circadian output behaviors with species specific relevance and differing sensitivity to various inputs. Interestingly, some basic concepts may be transferable although the chronobiological function substantially differs between species (e.g., sun compass orientation, time-place-learning, emergence, and diapause). Nevertheless, restricting the research to model organisms is insufficient to understand quite a few aspects in insect clock evolution. We may learn best from insect models, when we investigate the circadian clock in parallel in various insects, which display a modification or more pronounced function of the circadian clock, like diapause in northern flies or other insects with pronounced diapause [e.g., aphids (Barberà and Martínez-Torres, 2017; Barberà et al., 2017), bugs (Kotwica-Rolinska et al., 2017), wasps (Reznik, 2011; Paolucci et al., 2019), or butter flies (Denlinger et al., 2017)], or emergence rhythms in solitary bees. This may provide us furthermore with a better insight into circadian clock evolution. Even task related plasticity in the circadian clock is not restricted to honey bees. Ants were also found to perform arrhythmic brood care and have task related plasticity in clock gene expression (Ingram et al., 2009; Fujioka et al., 2017). This indicates, that although sociality evolved several times independently in Hymenoptera (in bees, wasps and ants), there seems to be a common ground plan to the social clock of Hymenoptera [reviewed in Bloch (2009), Bloch (2010), Bloch and Grozinger (2011)].

Studying modifications to the circadian clock in social and non-social Hymenopterans may be the key to understand the concept of social clocks. Especially bees (Anthophila) provide a huge range of differently scaled social lifestyles. We find true social (eusocial) bees (e.g., honey bees), primitively social (e.g., bumble bees), facultatively social and solitary bees (Shell and Rehan, 2018). Primitively social bumble bee queens also display circadian plasticity: when founding a new colony they take care of their first brood without behavioral rhythms, but resume rhythmic activity, when the brood is removed (Eban-Rothschild et al., 2011). Contrary to honey bees, brood care of bumble bee workers is rather related to their body size than their age (Yerushalmi et al., 2006).

Regarding the molecular clock, it appears not enough to compare the clockwork of social Hymenoptera with solitary insects from other orders (like for example *D. melanogaster*), because the unique composition of the circadian clock gene set in Hymenoptera indicates a regulatory mechanism that is different from the one in other insect orders. Therefore, exploring the clock genes, neuronal network and clock regulated behavior in solitary bees (and bees of different social grades) appears essential in future studies on the Hymenopteran clock.

Finally, we want to mention one further topic: inter-species interactions and co-evolution in chronobiology. Apart from temporal reproductive barriers (see above: species specific mating flights in honey bees), cohabitating insects establish species specific daytime-dependent foraging activity because of competition for food resources (Krell-Westerwalbesloh et al., 2004; Gottlieb et al., 2005; Wcislo and Tierney, 2009; Bloch et al., 2017; Smith et al., 2017). For example, the solitary bee *Proxylocopa olivieri* forages with a bimodal activity peaking at dusk and dawn and thereby avoids interaction with other bee species like *A. mellifera*, which shows unimodal foraging during the day (Gottlieb et al., 2005). Or in case of different cohabitating dung beetle guilds, the superior competitors are active during the day, while beetle guilds of lower competitive status display a peak in activity around dusk (Krell-Westerwalbesloh et al., 2004). Such inter-species effects in insect interaction networks, just like species specific clock outputs, clearly can only be sufficiently researched by studying the circadian clock of both, model and non-model insects in chronobiology.

## Author Contributions

All authors listed have made a substantial, direct and intellectual contribution to the work, and approved it for publication.

## Conflict of Interest

The authors declare that the research was conducted in the absence of any commercial or financial relationships that could be construed as a potential conflict of interest. The reviewer CK declared a past co-authorship with one of the authors CH-F to the handling Editor
